# Factors associated with 1-year visual response following intravitreal bevacizumab treatment for diabetic macular edema: a retrospective single center study

**DOI:** 10.1186/s40942-021-00286-9

**Published:** 2021-03-04

**Authors:** Janejit Choovuthayakorn, Apichat Tantraworasin, Phichayut Phinyo, Jayanton Patumanond, Paradee Kunavisarut, Titipol Srisomboon, Pawara Winaikosol, Direk Patikulsila, Voraporn Chaikitmongkol, Nawat Watanachai, Kessara Pathanapitoon

**Affiliations:** 1 Department of Ophthalmology, Faculty of Medicine, Chiang Mai University, Chiang Mai, Thailand; 2 Department of Surgery, Faculty of Medicine, Chiang Mai University, Chiang Mai, Thailand; 3 Clinical Epidemiology and Clinical Statistics Center, Faculty of Medicine, Chiang Mai University, Chiang Mai, Thailand; 4 Department of Family Medicine, Faculty of Medicine, Chiang Mai University, Chiang Mai, Thailand; 5 Musculoskeletal Science and Translational Research (MSTR), Chiang Mai University, Chiang Mai, Thailand

**Keywords:** Diabetic macular edema, Predictors, Bevacizumab

## Abstract

**Background:**

To explore the association of clinical characteristics and retinal microstructural features on optical coherence tomography in predicting 1-year visual response following intravitreal bevacizumab injections in eyes with visual impairment from center-involved diabetic macular edema (CI-DME).

**Methods:**

Medical records of patients with visual impairment from CI-DME, who initiated intravitreal bevacizumab injections between Jan 2012 and Dec 2016 and were followed for a minimum of 12 months were retrospectively reviewed.

**Results:**

The study included 226 eyes with a mean (SD) baseline visual acuity (VA) of 51.8 (19.1) letters. At week 12, following the three initial treatments, a mean (SD) VA improved to 61.7 (17.8) letters. Visual gain ≥ 10 letters was observed in 109 eyes (48.2%), while a limited early visual gain < 5 letters was noted in 80 eyes (35.4%). At one year, 110 eyes (48.7%) achieved a good VA gain ≥ 10 letters. In addition, eyes with poor baseline VA had a higher proportion of eyes that obtained limited early VA gained at week 12 (< 5 letters) and maintained in this visual response category at moth 12 compared to eyes with better baseline VA (74.1% versus 59.1%). In the multivariable logistic regression, the following factors reduced the probability of 1-year visual gain ≥ 10 letters: elderly (p = 0.040), better baseline vision (p = 0.001), and limited early visual gain < 5 letters at week 12 (p < 0.001). In multivariable linear regression, male (p = 0.010) and eyes with the presence of hyperreflective foci on baseline OCT (p = 0.010) were likely to have higher VA improvement. However, eyes with better baseline VA (p = 0.002), limited early VA gain at week 12 (p < 0.001), and a presence of EZ disruption at week 12 (p = 0.002) were likely to have less VA improvement.

**Conclusions:**

Although bevacizumab is considered as effective management for CI-DME, variability in treatment responses is expected. This study revealed that baseline characteristics and visual responses at week 12 might help predict the long-term treatment response. Eyes with characteristics at risk of limited long-term visual outcome may require attention in optimizing their individual treatment strategies.

## Introduction

Diabetic macular edema (DME) is one of the leading causes of central visual impairment in diabetic patients. There are several pathophysiologic and biochemical changes contributing to the development of DME; however, an increase in vascular endothelial growth factor (VEGF) has been reported as a potent related mediator [1, 2]. In a previous publication, a significantly higher serum VEGF level was found in diabetic patients with more severe diabetic retinopathy (DR), and more severe disruption of photoreceptor outer segments (external limiting membrane (ELM) and ellipsoid zone (EZ)), compared to less severe DR patients and healthy controls. Moreover, a positive association between grades of photoreceptor outer segment disruption and the degree of visual acuity (VA) reduction was also observed [3]. Based on randomized clinical trials (RCTs), the remarkable improvement in visual and anatomical outcomes following intravitreal anti-VEGF injection for visual impairment from center-involved DME (CI-DME), compared to macular photocoagulation, has been reported [4–7]. This significant efficacy was highlighted across all three available anti-VEGF agents (bevacizumab, ranibizumab, and aflibercept) and treatment regimens that were used. However, lower visual gains were noted in a poor baseline VA patient treated with bevacizumab [8–10]. Consequently, intravitreal anti-VEGF injection becomes a mainstay treatment option for CI-DME. Despite these improvements, variations in individualized treatment responses have been observed in both RCTs and the real-world clinical settings [11–13].

Due to financial burden, bevacizumab has been administered as the first-line anti-VEGF agent in several clinical practices regardless of baseline VA and mostly with a fewer number of injections than in RCT-derived protocols. The treatment response pattern in these clinical settings is necessary for evaluating optimal management. Determining associated factors for visual outcomes following intravitreal bevacizumab injection in clinical practice for CI-DME may influence the patients’ expectations and the physicians’ treatment decisions to adjust the therapeutic regimens and their intensity, as well as to consider the alternative therapeutic modalities.

Therefore, this study primarily aimed to explore demographics, clinical characteristics, and optical coherence tomography (OCT) features at baseline and at week 12 (early response) that may be associated with a 1-year visual response following intravitreal bevacizumab injections in eyes presenting with visual impairment from CI-DME. The results may provide additional information for a less intense intravitreal bevacizumab treatment for CI-DME in a real-world setting.

## Material and methods

This retrospective observational study was approved by the Research Ethics Committee, Faculty of Medicine, Chiang Mai University. The protocol was performed in accordance with the Declaration of Helsinki and its later amendments. Informed consent was waived due to anonymous data extraction with no direct patient and public involvement in the study.

### Study participants

The medical records of consecutive patients who had visual impairment from CI-DME, diagnosed by clinical examination and confirmed by spectral-domain optical coherence tomography (SD-OCT), started the first intravitreal anti-VEGF treatment between January 2012 and December 2016, were identified. Eligible patients were those who met all the following criteria: (1) received three initial monthly consecutive loading injections; (2) diagnosed with type 1 or type 2 diabetes; (3) had initial VA of 20/32 or worse; (4) had CI-DME defined as an average macular thickness of 1-mm diameter circle centered at the fovea (central subfield thickness, CSFT) measured by OCT ≥ 320 µm; (5) had VA and OCT data at baseline and 12 weeks after three consecutive injections; and (6) had a follow-up of at least 12 months after the first injection. Excluded from the study were eyes with any of the following conditions: (1) had concomitant ocular diseases that would impact macular thickness and VA interpretation; (2) had a history of vitreoretinal surgery or underwent within the study period; (3) had undergone cataract surgery within four months prior to anti-VEGF injection or during the study period; (4) had undergone macular laser photocoagulation within three months prior to anti-VEGF injection; (5) had significant epiretinal membrane and traction that preclude the benefit of intravitreal anti-VEGF injection by physician discretion; (6) had received intravitreal steroid injection within four months before initiating intravitreal anti-VEGF injection or within the study period; (7) had a history of chronic kidney disease requiring dialysis; or (8) administered any systemic anti-VEGF medications within six months prior to anti-VEGF injection or during the study period. Both eyes of patients who received bilateral anti-VEGF injections were included. Following three initial monthly loading injections, subsequent treatments were administered in eyes with non-stability in VA and/or CSFT (defined as changes in VA ≥ 1 Snellen line or changes in CSFT ≥ 10% compared to the previous visit). The injection was withheld in stabilized eyes, and the next visit was extended from 4 to 8 weeks. If the stabilization was secured in the subsequent visit, the injection was, again, withheld, and the next visit was further extended to 16 weeks. Re-injections were performed in cases of worsening of VA or CSFT. Based on physician discretion, macular laser photocoagulation was considered in eyes with stability in VA and CSFT, but still had persistent macular thickening.

At baseline, patients’ demographics including age, gender, duration of diabetes and blurred vision, associated systemic diseases, diabetic retinopathy staging, and history of previous diabetic retinopathy and/or DME treatments were collected. In addition, at baseline and each follow-up visit, ocular characteristics including VA, anterior and posterior segment findings by slit-lamp examination, intraocular pressure, quantitative CSFT value measured by OCT, intravitreal anti-VEGF injection, and additional macular laser photocoagulation were also reviewed.

### Optical coherence tomography imaging

The OCT images were obtained using Spectralis HRA SD-OCT (Heidelberg Engineering, GmbH, Heidelberg, Germany) with raster scans over the macular area by 20° × 20°. Each horizontal B-scan consisted of 512 A-scans density and were averaged by nine automatic real-time images. The automatic retinal tracking was operated to ensure the exact scanned retinal locations at each follow-up visit. The CSFT values calculated by incorporated machine software were recorded. Three horizontal B-scans (one B-scan passing through the fovea and 2 B-scans located 500 µm above and below the fovea) were qualitatively assessed within central 1-mm central subfield for specific retinal morphological features. The patterns of macular edema including diffuse and intraretinal cyst (IRC), characterized as intraretinal round or oval low reflectivity cystoid-like spaces, were identified. Additionally, the presence of IRC with a horizontal diameter of ≥ 600 µm was further categorized. For the inner retinal layer, the disorganization of retinal inner layer (DRIL), defined as the disruption of demarcation junction between ganglion cell layer/inner plexiform layer and inner nuclear layer/outer plexiform layer, involving more than 50% of the scanned area was assessed [14]. For the outer retinal layer, the disruption of the external limiting membrane (ELM) and ellipsoid zone (EZ) involving more than 50% of the scanned area was also determined. Presence of subretinal fluid (SRF) was characterized by optically clear space between the sensory retina and the retinal pigment epithelium. According to the International Vitreomacular Traction Study Group, vitreoretinal interface abnormalities were assessed for the presence of epiretinal membrane (ERM) [15]. The number of intraretinal hyperreflective foci, sized < 30 µm with no back-shadowing, were counted and further classified as having < 30 vs ≥ 30 foci. Presence of subfoveal exudate was also evaluated. All OCT retinal morphologies were graded at baseline and week 12 following the first three consecutive monthly injections by two independent graders masked to clinical information (TS and PW). In case of disagreement, differences were solved by discussion.

### Statistical analysis

Based on a preliminary review of 40 medical records, the mean (SD) VA difference from baseline was 11.9 (15.6) and 4.3 (10.7) in patients with baseline VA < 69 letters and ≥ 69 letters, respectively. Using the two-sample comparison of means, a total of 98 eyes (49 in each baseline VA group) was needed to provide 80% statistical power with a two-sided alpha error of 0.05.

Demographic characteristics were presented by descriptive analysis (mean and standard deviation (SD) for continuous data and percentage for categorical data). Snellen VA was converted to approximate early treatment diabetic retinopathy study (approxETDRS) letter scores for statistical analysis. At week 12, the proportion of eyes that experienced VA gain ≥ 10 and < 5 letters, eyes that had a reduction in CSFT ≥ 10%, and microstructural changes on OCT compared to baseline were evaluated. Also, at the 1-year visit, a proportion of eye gaining VA ≥ 10 letters (good VA gain) and a mean change in VA from baseline were estimated. To control the association between datasets, generalized estimating equation (GEE) was used. Multivariable analysis for factors related to status of 1-year visual improvement ≥ 10 letters was performed by binary logistic regression. In addition, multivariable analysis for factors related to 1-year mean VA change from baseline was performed by generalized linear regression. Independent variables in each model were derived from the univariable analysis with the significant association. Additionally, number of injections, and additional macular laser photocoagulation were also adjusted in the models. Correlations between VA and CSFT were calculated using the Spearman correlation coefficient. Data analysis was performed by STATA version 16, and a p value less than 0.05 was considered statistically significant.

## Results

Among 310 eyes which completed three initial bevacizumab treatments, 84 eyes were excluded due to no OCT images at week 12 and/or at month 12 (38 eyes), switching to other anti-VEGF treatments (25 eyes), undergoing cataract extraction (5 eyes), and low OCT quality images (16 eyes) (Additional file 1: Fig. S1). In total, 226 eyes (of 173 patients) with a mean (SD) age of 57.5 (8.6) years, were included in the analysis. Eighty patients (80/173, 46.2%) were female. Overall, the study eyes had a mean (SD) baseline VA of 51.8 (19.1) letters (Snellen equivalent 20/100) and a mean (SD) baseline CSFT of 496.2 (145.2) µm. Sixty-three eyes (63/226, 27.9%) had a good baseline VA (≥ 69 letters), and 163 eyes (72.1%) had a poor baseline VA (< 69 letters). All eyes received intravitreal bevacizumab treatments during the entire 12 observed months with a mean (SD) of 7.3 (3.2) injections.

### Visual responses during 1-year follow-up period

**Fig. 1:**
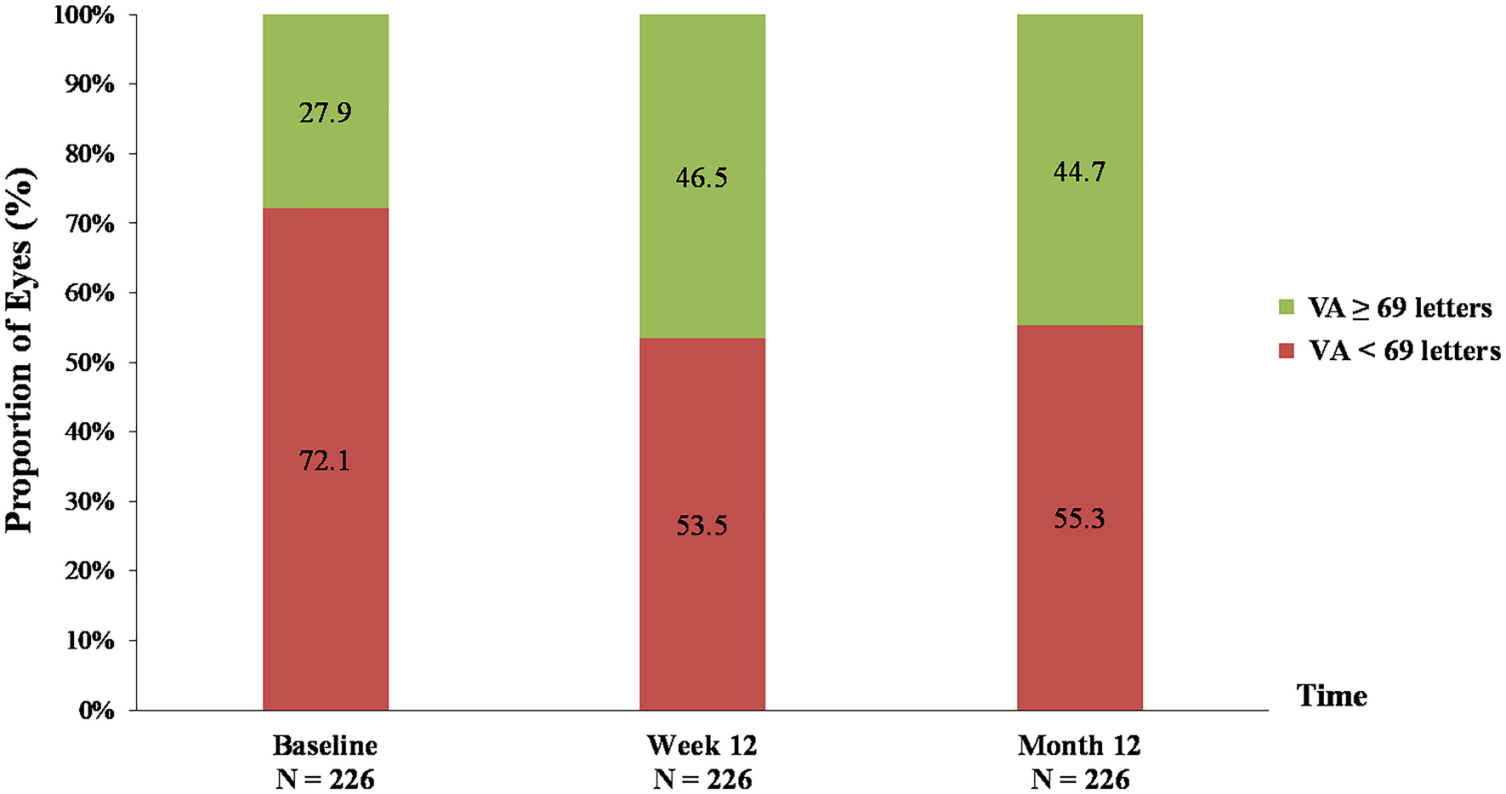
Proportion of eyes stratified by visual acuity level at baseline, week 12, and month 12 following intravitreal bevacizumab injections for visual impairment from center-involved diabetic macular edema

At week 12 (after three initial monthly intravitreal treatments), the mean (SD) VA improved to 61.7 (17.8) letters (Snellen equivalent 20/60). Among these, 109 eyes achieved a good VA gain of ≥ 10 letters (20/63 (31.8%) eyes with good baseline VA and 89/163 (54.6%) eyes with poor baseline VA). A visual gain of < 5 letters was observed in 80 eyes (22/63 (34.9%) eyes with good baseline VA and 58/163 (35.6%) eyes with poor baseline VA). At one year, the overall mean (SD) VA was 61.2 (18.4) letters (Snellen equivalent 20/60), and 110 eyes (48.7%) obtained VA gain of ≥ 10 letters. The proportions of eyes that gained < 5 letters at 12 weeks and remained in the same group at one year were 13/22 with good baseline VA (59.1%) and 43/58 with poor baseline VA (74.1%). The distribution of VA levels over the study period are shown in Fig. 1. Demographics and characteristics of treated eyes stratified by 1-year VA improvements and 1-year mean change in VA are summarized in Table 1 and Additional file 2: Table S1, respectively.

**Table 1:** Demographics, characteristics, and interventions of eyes with visual impairment from center-involved diabetic macular edema stratified by visual acuity response at one year following treatments

Characteristics	Overall (N = 226 Eyes)	Gain ≥ 10 Letters at 1 Year (N = 110 Eyes)	Gain < 10 Letters at 1 Year (N = 116 Eyes)	P Value*
Demographics				
Age ≥ 60 years, n (%)	78 (34.5)	30 (27.3)	48 (41.4)	0.041
Male, n (%)	122 (54)	66 (60)	56 (48.3)	0.112
Severe NPDR to PDR stage, n (%)	158 (69.9)	75 (68.2)	83 (71.6)	0.638
Previous macular laser photocoagulation, n (%)	42 (18.6)	13 (11.8)	29 (25)	0.019
Previous PRP, n (%)	63 (27.9)	28 (25.5)	35 (30.2)	0.528
Phakia, n (%)	202 (89.4)	102 (92.7)	100 (86.2)	0.115
Ocular characteristics at baseline				
Mean (SD) VA, letter	51.8 (19.1)	46 (21)	57.3 (15.2)	< 0.001
VA ≥ 69 letters, n (%)	63 (27.9)	23 (20.9)	40 (34.5)	0.025
Mean CSFT (SD), µm	496.2 (145.2)	513.9 (164.5)	479.4 (122.6)	0.159
CSFT ≥ 400 µm	163 (72.1)	80 (72.3)	83 (81.6)	0.983
ERM, n (%)	28 (12.4)	7 (6.4)	21 (18.1)	0.012
DRIL, n (%)	65 (28.8)	24 (21.8)	41 (35.3)	0.026
HF, n (%)	99 (43.8)	52 (47.3)	47 (40.5)	0.257
IRC ≥ 600 µm, n (%)	23 (10.2)	9 (8.2)	14 (12.1)	0.399
Disruption of ELM, n (%)	61 (26.9)	30 (27.3)	31 (26.7)	0.858
Disruption of EZ, n (%)	56 (24.8)	18 (16.4)	38 (32.8)	0.003
SRF, n (%)	113 (50)	59 (53.6)	54 (46.6)	0.316
Foveal exudate, n (%)	15 (6.6)	6 (5.5)	9 (7.8)	0.507
Ocular characteristics at week 12				
Mean (SD) VA, letter	61.7 (17.8)	63.6 (17.8)	59.9 (17.7)	0.172
VA gain < 5 letters, n (%)	80 (35.4)	13 (11.8)	67 (57.8)	< 0.001
CSFT reduction < 10%, n (%)	78 (34.5)	27 (24.6)	51 (44.0)	0.004
DRIL, n (%)	42 (18.6)	13 (11.8)	29 (25)	0.012
HF, n (%)	63 (27.9)	31 (28.2)	32 (27.6)	0.812
IRC ≥ 600 µm, n (%)	15 (6.6)	4 (3.6)	11 (9.5)	0.046
Disruption of ELM, n (%)	43 (19)	12 (10.9)	31 (26.7)	0.002
Disruption of EZ, n (%)	44 (19.5)	12 (10.9)	32 (27.6)	0.002
SRF, n (%)	41 (18.1)	24 (21.8)	17 (14.7)	0.256
Foveal exudate, n (%)	17 (7.5)	6 (5.5)	11 (9.5)	0.428
During 12 months				
Mean (SD) number of injections	7.3 (3.2)	6.9 (3.2)	7.5 (3.2)	0.183
Receiving additional macular laser photocoagulation, n (%)	127 (56.2)	59 (53.6)	68 (58.6)	0.494

*VA * visual acuity,*SD* standard deviation, *NPDR*  *non-proliferative diabetic retinopathy,PDR* *proliferative diabetic retinopathy, PRP*  *panretinal photocoagulation, CSFT* *central subfield thickness, ERM*  *epiretinal membrane, DRIL* *disorganization of retinal inner layer, HF* *hyperreflective foci, IRC* *intraretinal cyst, ELM* *external limiting membrane, EZ* *ellipsoid zone, SRF* *subretinal fluid*

*generalized estimating equation (GEE)

### Optical coherence tomographic characteristics

For the baseline OCT features, although there were no significant differences in CSFT between 1-year VA improvement groups, eyes with 1-year VA gain of ≥ 10 letters had significantly less proportion of ERM (p = 0.012), less proportion of DRIL (p = 0.026), and less disruption of EZ (p = 0.003), compared to eyes with VA gain < 10 letters at one year.

At week 12, the restoration of retinal microstructures was observed in all study eyes. However, eyes with 1-year VA gain of ≥ 10 letters had significantly less proportion of the following OCT characteristics: DRIL (p = 0.012), presence of IRC sized ≥ 600 µm (p = 0.046), disruption of ELM (p = 0.002), and disruption of EZ (p = 0.002) compared to eyes with VA gain < 10 letters at one year. Details of OCT characteristics at baseline and week 12 following treatments stratified by 1-year VA improvement groups are described in Table 1. For the 1-year mean change in VA, the overall mean improvement was lower in eyes with good baseline VA than with poor baseline VA (4.6 vs 11.1 letters, p < 0.001). Details of related factors at baseline and week 12 following treatments to 1-year mean VA change are shown (Additional file 2: Table S1).

### Correlation between VA and CSFT

**Fig. 2:**
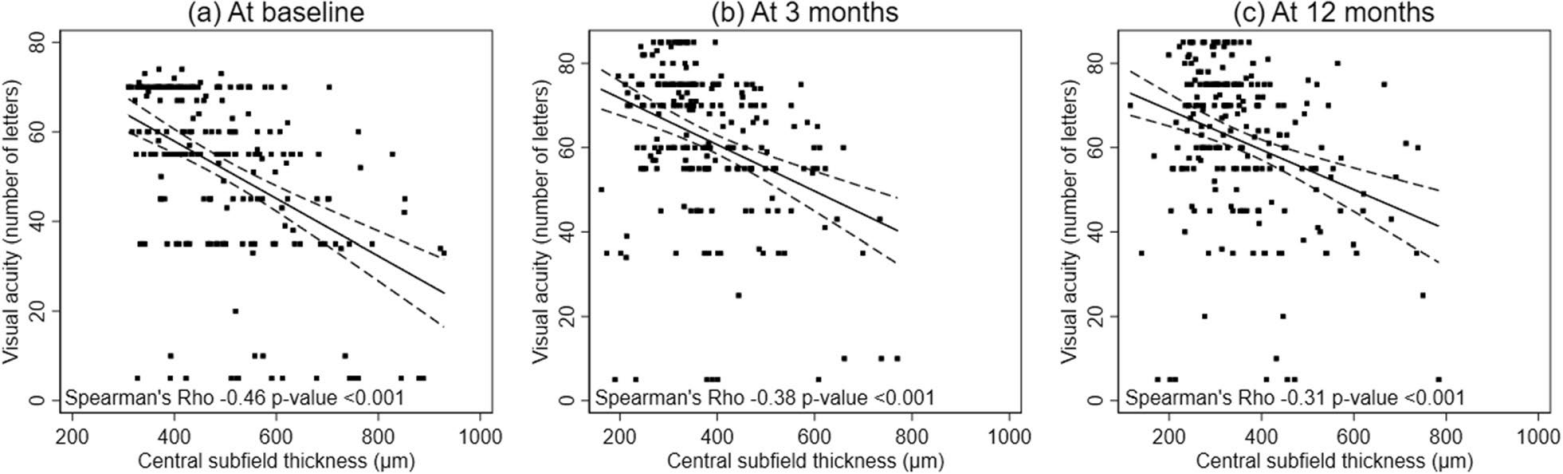
Correlation between visual acuity and central subfield thickness for eyes with visual impairment from center-involved diabetic macular edema following intravitreal bevacizumab injections over a study period: **a** at baseline; **b** at 3 months; and **c** at 12 months

A negative correlation between VA and CSFT was demonstrated by a decreasing correlation coefficient over time (− 0.46 at baseline, − 0.38 at month 3 after 3 loading injections, and − 0.31 at 1 year of treatments) with all p values < 0.001 (Fig. 2).

### Multivariable analysis for 1-year VA change

**Fig. 3:**
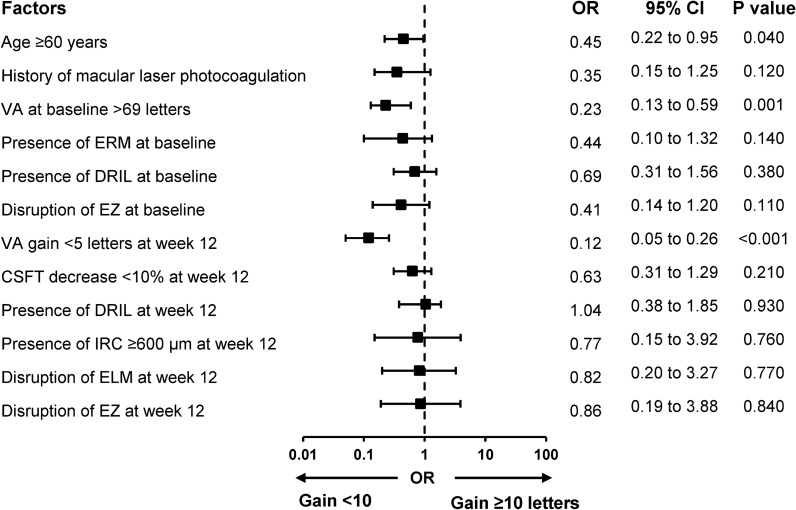
Multivariable logistic regression analysis for factors related to a good vision gain at one year for eyes with visual impairment from center-involved diabetic macular edema following treatments. VA Visual acuity, ERM Epiretinal membrane, DRIL Disorganization of retinal inner layer, EZ Ellipsoid zone, CSFT Central subfield thickness, IRC Intraretinal cyst, ELM External limiting membrane

By multivariable logistic regression, patients with the following factors: an older age (odds ratio: 0.45, 95% confidence interval (CI): 0.22 to 0.95, p = 0.040); a better baseline VA (odds ratio: 0.23, 95% CI 0.13 to 0.59, p = 0.001); and a limited VA gain at week 12 (odds ratio: 0.12, 95% CI 0.05 to 0.26, p < 0.001) were less likely to have visual improvement ≥ 10 letters at one year (Fig. 3). Moreover, multivariable linear regression additionally demonstrated that male and presence of hyperreflective foci on baseline OCT had positive association for mean VA improvement (coefficient: 4.86 letters, 95% CI 1.31 to 8.41, p = 0.010 and coefficient: 3.59 letters, 95% CI 1.58 to 7.65, p = 0.010, respectively). On the contrary, a better baseline VA, a limited VA gain at week 12, and a presence of EZ disruption at week 12 had negative association for mean VA improvement ([coefficient: − 5.63 letters, 95% CI − 9.75 to -2.52, p = 0.002], [coefficient: − 10.38, 95% CI − 12.31 to − 7.45, p < 0.001], and [coefficient: − 10.83 letters, CI − 18.37 to -3.30, p = 0.002], respectively) (Additional file 3: Fig S2).

## Discussion

In this report, with an overall improvement in mean VA, approximately half of the study eyes achieved a good visual gain of ≥ 10 letters at the end of one year. More than half of eyes that gained VA < 5 letters at week 12 remained in the same visual response group at one year. The significant predictors to determine the probability of 1-year visual improvement and/or 1-year mean VA change were age, gender, baseline VA, baseline hyperreflective foci on OCT, an early visual response observed at week 12 following three initial injections, and disruption of EZ on OCT at week 12.

In the past, several biomarkers for predicting baseline visual impairment as well as visual responses following anti-VEGF treatment in eyes with CI-DME have been investigated [16–21]. Among these, quantitative retinal thickness data from OCT has become commonly used as an indicator for decision making and/or monitoring disease progression in RCTs and clinical practice [4, 6, 7, 11, 12]. However, several investigators have demonstrated the discrepancies in the relationship between CSFT and VA in DME [21–24]. Ou and associates reported a significant medium negative correlation between VA and CSFT at baseline and between changes in VA and CSFT at month 12 (r = − 0.42 and − 0.45, respectively). On the contrary, Gerendas and associates documented a weak negative correlation (r = − 0.34) of VA and CSFT at baseline with a further reduction in correlation (r = − 0.26) at 3 years following intravitreal anti-VEGF treatment for DME. The findings were in accordance with a hypothesis that not only CSFT, but also other factors are involved in visual responses following DME treatment.

With a better image resolution of subsequently developed SD-OCT, the associations between quantitative and/or qualitative retinal microstructures on SD-OCT and visual outcomes following anti-VEGF treatment for CI-DME have been extensively explored [25–30]. Chung and associates reported that the preservation of ELM and EZ integrity were associated with a better baseline VA and visual outcomes after one intravitreal bevacizumab injection [20]. Faran and associates found that the presence of baseline retinal tissue bridging between inner and outer retinal layers of DME eyes presenting with intraretinal cystoid cavities was a good predictor for visual recovery when followed for at least six months [31]. Likewise, baseline DRIL, SRF, and hyperreflective foci in the outer retinal layers have also been shown as the predictors for visual responses at one year following anti-VEGF treatment in other studies [32, 33]. Apart from these baseline OCT features, the subsequent clinical and structural OCT changes after anti-VEGF treatment have also been reported as the predictors for visual responses for eyes with CI-DME [34–38]. Sun and associates have shown a reduction in DRIL extent at four months, indicating the reversibility of inner retinal cells function, related to better VA at 12 months [14]. Other publications have reported that VA and anatomical response at 12 weeks after intravitreal anti-VEGF injections were potential factors for predicting long term visual outcomes [34–38].

This exploratory study revealed the association of age, gender, presenting VA, baseline hyperreflective foci, 12-week EZ integrity, and 12-week visual response with 1-year visual improvements following intravitreal bevacizumab injections. Younger age had been documented as a predictor for good visual gain following anti-VEGF treatment for DME in several studies even though the mechanisms are uncertain [17, 19, 39]. However, it may partly refer to the greater capability of the blood-retinal barrier to maintain its integrity and restore function in younger than the older patients. Analysis from RISE/RIDE data also showed that male gender was one of baseline predictors for VA ≥ 20/40 at the end of year 2 in ranibizumab-treated eyes. Similarly, male gender was also found as an associated factor for better long-term visual outcome in this study. With limited evidence, the relationship between gender and VA response in DME may require further investigation.

An impact of baseline VA level to visual improvement following anti-VEGF treatments in DME has been evidenced in several reports [40, 41]. This study reported a higher probability of eyes with poorer baseline VA to achieve significant VA improvements. This association may be partly explained by the floor effect (a tendency of eyes with poor baseline VA to have more gap for VA improvement). Baseline VA level also related to early visual responses. The results showed that a proportion of eyes in the limited early visual response that remained in this category at 1-year was higher in a poor baseline VA group than a group of better baseline VA. However, the explanation may partly relate to a lower mean number of injections in this study compared to the RCTS (7 vs 8 to 9 injections). The impact of higher treatment intensity to a more meaningful visual improvement in DME has been reported in real-world studies [42, 43]. Therefore, an increase in treatment frequency with the same anti-VEGF medication in other clinical settings may result in different treatment response patterns. Even with lacking comparative data, a treatment regimen adjustment or switching to more potent anti-VEGF agents/other alternative therapies may consider for poor baseline VA patients with limited early visual response.

The association of baseline hyperreflective foci on OCT and better visual improvement following intravitreal bevacizumab treatment for DME in this study was consistent with previous study [33, 44]. Inhibition of microglia/macrophage activation as well as a recovery of photoreceptors damaged by anti-VEGF, possibly explain this association. The characteristics of baseline EZ on OCT have also been shown as a potent factor for visual gain following DME treatment [45, 46]. De and associates reported an association of ELM and EZ restoration (a complete restoration of ELM preceding a complete restoration of EZ) with VA improvement after three initial intravitreal bevacizumab injections for DME [47]. According to previous report, this study found that eyes with persistent disruption of EZ after the loading phase were less likely to have visual improvement at 1-year. The result supports the importance of an intact EZ on OCT with visual gain, which may reflect the integrity of the photoreceptor inner segment, mainly occupied by the mitochondria, in the ellipsoid zone, and the preservation of visual transmission. However, the impact of differences in current OCT segmentation and measurement among studies should be considered. The development of a consensus on OCT features classification and the invention of an automated retinal grading system may decrease these OCT biomarker variabilities and facilitate their clinical applications [48–50].

The limitation of this study was the retrospective design with some unavailable information including duration of DM and/or DME, associated systemic diseases, angiographic data, and retinal microstructural changes after each treatment. These factors could also contribute to the DME treatment response patterns of anti-VEGF and long-term visual outcomes. In addition, due to the real-world clinical settings in this study, treatment and re-treatment criteria (that being adjudicated after the loading phase), and the frequency of treatments may differ from other clinical settings. Therefore, the clinical applications of the data should be taken into consideration. However, this study supports evidence for visual improvement and predictors for long-term visual outcomes where bevacizumab is the main treatment option in clinical settings.

## Conclusion

In CI-DME eyes, baseline parameters including young age, male gender, lower VA level, and presence of hyperreflective foci are related with remarkable VA improvement following intravitreal bevacizumab injections. In addition, early visual response and maintenance of EZ are also beneficial factors in predicting a long-term visual response. In managing eyes with CI-DME, these prognostic factors should be taken into consideration when adjusting the therapeutic strategies.

## Supplementary Information

**Additional file 1: Fig. S1.** Flow diagram for inclusion and exclusion of eyes with visual impairment from center-involved diabetic macular edema.

**Additional file 2: Table. S1.** Univariable analysis for mean change in visual acuity from baseline at one year of eyes with visual impairment from center-involved diabetic macular edema

**Additional file 3: Fig. S2.** Multivariable linear regression analysis for factors related to mean change in vision at one year for eyes with visual impairment from center-involved diabetic macular edema following treatments. *VA* visual acuity,* ERM *epiretinal membrane,* DRIL* disorganization of retinal inner layer,* EZ* ellipsoid zone,* CSFT *central subfield thickness,* EZ* ellipsoid zone,* ELM* external limiting membrane.

## Data Availability

The datasets used and/or analyzed in this study are available from the corresponding author on reasonable request.

## Abbreviations

approxETDRS: Approximate early treatment diabetic retinopathy study
CI-DME: Center-involved diabetic macular edema
CSFT: Central subfield thickness
DME: Diabetic macular edema
DRIL: Disorganization of retinal inner layer
ELM: External limiting membrane
ERM: Epiretinal membrane
EZ: Ellipsoid zone
GEE: Generalized estimating equation
IRC: Intraretinal cyst
OCT: Optical coherence tomography
RCTs: Randomized clinical trials
SD: Standard deviation
SD-OCT: Spectral domain optical coherence tomography
SRF: Subretinal fluid
VA: Visual acuity
VEGF: Vascular endothelial growth factor
