# Supplementary figures and images for: Factors associated with 1-year visual response following intravitreal bevacizumab treatment for diabetic macular edema: a retrospective single center study
Source: Int J Retina Vitreous. 2021 Mar 4;7:17. doi: 10.1186/s40942-021-00286-9 (PMC7931592; doi:10.1186/s40942-021-00286-9)

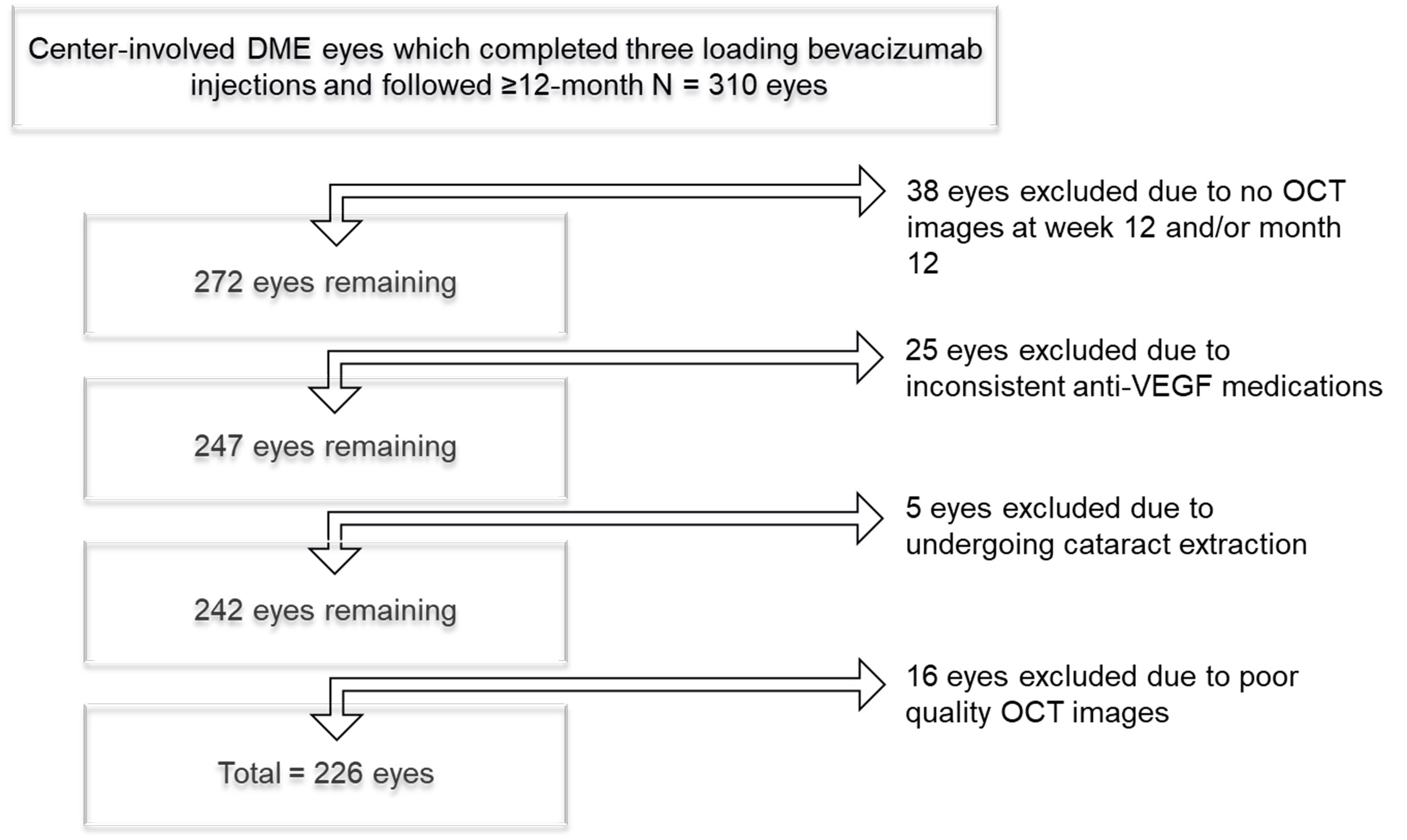

Supplement: Supplementary file 1 — Additional file 1: Fig. S1. Flow diagram for inclusion and exclusion of eyes with visual impairment from center-involved diabetic macular edema. [file 40942_2021_286_MOESM1_ESM.jpg]

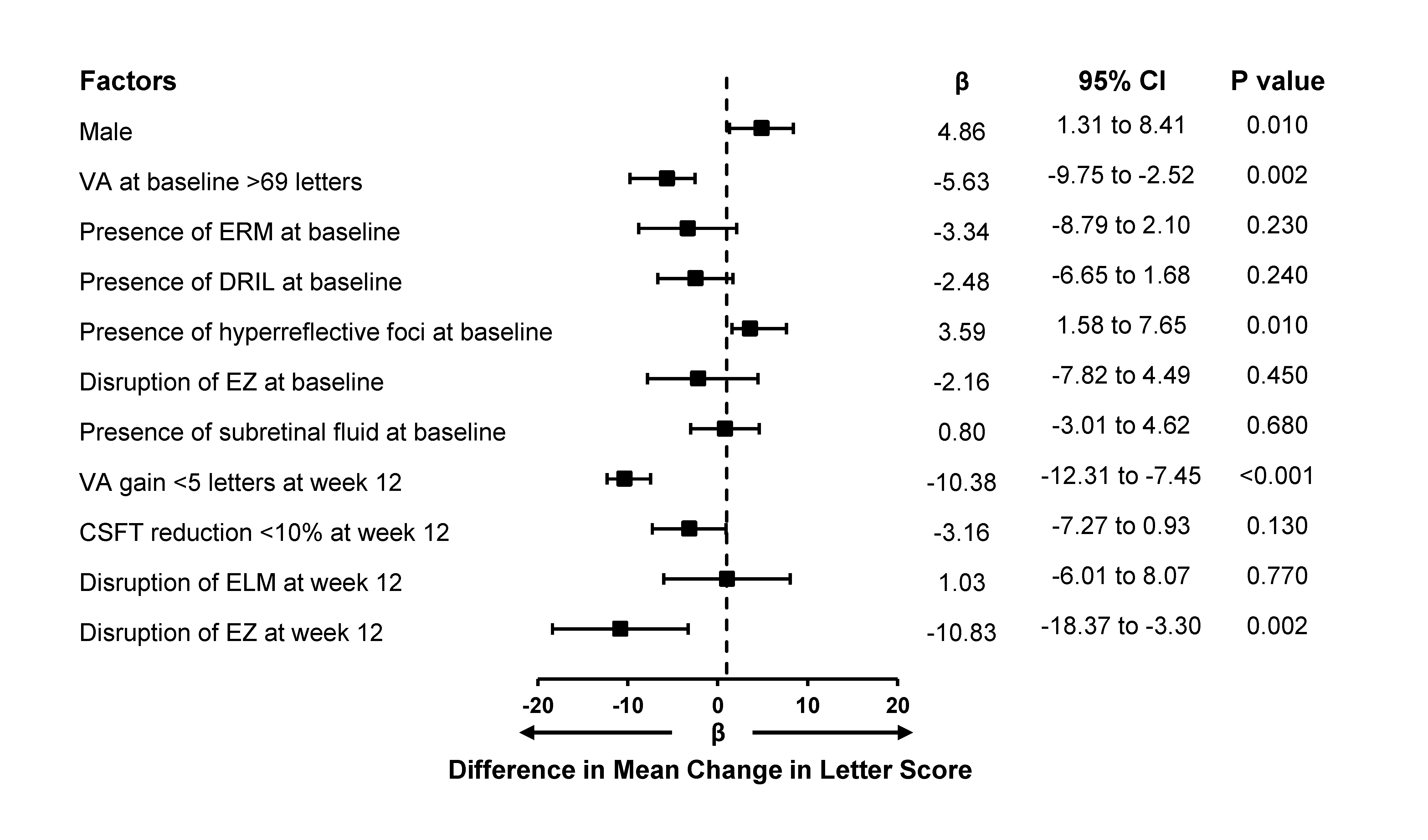

Supplement: Supplementary file 3 — Additional file 3: Fig. S2. Multivariable linear regression analysis for factors related to mean change in vision at one year for eyes with visual impairment from center-involved diabetic macular edema following treatments. VA visual acuity, ERM epiretinal membrane, DRIL disorganization of retinal inner layer, EZ ellipsoid zone, CSFT central subfield thickness, EZ ellipsoid zone, ELM external limiting membrane. [file 40942_2021_286_MOESM3_ESM.png]
