# Supplementary material for: Factors associated with 1-year visual response following intravitreal bevacizumab treatment for diabetic macular edema: a retrospective single center study
Source: Int J Retina Vitreous. 2021 Mar 4;7:17. doi: 10.1186/s40942-021-00286-9 (PMC7931592; doi:10.1186/s40942-021-00286-9)
Supplement: Supplementary file 2 — Additional file 2: Table. S1. Univariable analysis for mean change in visual acuity from baseline at one year of eyes with visual impairment from center-involved diabetic macular edema [file 40942_2021_286_MOESM2_ESM.docx]

**Table 2**: Univariable analysis for mean change in visual acuity from baseline at one year of eyes with visual impairment from center-involved diabetic macular edema

| **Characteristics** | **N**  **(226 Eyes)** | **Change in Letter Scores at 1 Year,**  **Coefficient (95% CI)** | **P Value*** |
| --- | --- | --- | --- |
| **Demographics** |  |  |  |
| Age ≥60 years | 78 | -2.67 (-7.40 to 2.06) | 0.268 |
| Male | 122 | 5.45 (0.99 to 9.91) | 0.017 |
| Severe NPDR to PDR stage | 158 | 2.14 (-2.79 to 7.06) | 0.396 |
| Previous macular laser photocoagulation | 42 | -5.04 (-10.65 to 0.57) | 0.078 |
| Previous PRP | 63 | -3.25 (-8.20 to 1.70) | 0.198 |
| Phakia | 202 | 3.93 (-3.10 to 10.96) | 0.273 |
| **Ocular characteristics at baseline** |  |  |  |
| VA ≥69 ETDRS letter | 63 | -6.55 (-11.24 to -1.85) | 0.006 |
| CSFT ≥400 µm | 163 | 3.59 (-1.17 to 8.35) | 0.139 |
| ERM | 28 | -7.74 (-14.11 to -1.36) | 0.017 |
| DRIL | 65 | -5.18 (-9.90 to -0.47) | 0.031 |
| HF | 99 | 5.17 (0.83 to 9.50) | 0.020 |
| IRC ≥600 µm | 23 | -1.99 (-8.99 to 5.01) | 0.577 |
| ELM | 61 | -1.18 (-6.03 to 3.66) | 0.632 |
| EZ | 56 | -9.44 (-14.27 to -4.60) | <0.001 |
| SRF | 113 | 4.55 (0.35 to 8.75) | 0.034 |
| Foveal exudate | 15 | -1.52 (-10.16 to 7.12) | 0.731 |
| **Ocular characteristics at week 12** |  |  |  |
| VA gain <5 letters | 80 | -12.55 (-18.59 to -10.51) | 0.044 |
| CSFT reduction <10% | 78 | -8.04 (-12.40 to -3.68) | <0.001 |
| DRIL | 42 | -4.956 (-10.44 to 0.52) | 0.077 |
| HF | 63 | 2.35 (-2.35 to 7.05) | 0.327 |
| IRC ≥600 µm | 15 | -5.37 (-13.92 to 3.17) | 0.218 |
| ELM | 43 | -10.85 (-16.13 to -5.57) | <0.001 |
| EZ | 44 | -13.43 (-18.57 to -8.29) | <0.001 |
| SRF | 41 | 3.99 (-1.60 to 9.58) | 0.162 |
| Foveal exudate | 17 | -2.29 (-10.44 to 5.86) | 0.582 |
| *CI = confidence interval, VA = visual acuity, ETDRS = the Early Treatment Diabetic Retinopathy Study, NPDR = non-proliferative diabetic retinopathy, PDR = proliferative diabetic retinopathy, PRP = panretinal photocoagulation, CSFT = central subfield thickness, IRC = intraretinal cyst, ERM = epiretinal membrane, DRIL = disorganization of retinal inner layer, HF = hyperreflective foci, ELM = external limiting membrane, EZ = ellipsoid zone, SRF = subretinal fluid*  *Note: *generalized estimating equation (GEE)* | | | |
